# Patterns of linkage disequilibrium and association mapping in diploid alfalfa (*M. sativa* L.)

**DOI:** 10.1007/s00122-012-1854-2

**Published:** 2012-04-05

**Authors:** Muhammet Sakiroglu, Sue Sherman-Broyles, Alec Story, Kenneth J. Moore, Jeffery J. Doyle, E. Charles Brummer

**Affiliations:** 1 Department of Bioengineering, Kafkas University, Kars, 36100 Turkey; 2 Department of Plant Biology, Cornell University, Ithaca, NY 14853 USA; 3 Department of Agronomy, Iowa State University, Ames, IA 50011 USA; 4 Samuel Roberts Noble Foundation, 2510 Sam Noble Parkway, Ardmore, OK 73401 USA

## Abstract

Association mapping enables the detection of marker-trait associations in unstructured populations by taking advantage of historical linkage disequilibrium (LD) that exists between a marker and the true causative polymorphism of the trait phenotype. Our first objective was to understand the pattern of LD decay in the diploid alfalfa genome. We used 89 highly polymorphic SSR loci in 374 unimproved diploid alfalfa (*Medicago sativa* L.) genotypes from 120 accessions to infer chromosome-wide patterns of LD. We also sequenced four lignin biosynthesis candidate genes (*caffeoyl*-*CoA 3*-*O*-*methyltransferase* (*CCoAoMT*), *ferulate*-*5*-*hydroxylase* (*F5H*), *caffeic acid*-*O*-*methyltransferase* (*COMT*), and *phenylalanine amonialyase* (*PAL 1*)) to identify single nucleotide polymorphisms (SNPs) and infer within gene estimates of LD. As the second objective of this study, we conducted association mapping for cell wall components and agronomic traits using the SSR markers and SNPs from the four candidate genes. We found very little LD among SSR markers implying limited value for genomewide association studies. In contrast, within gene LD decayed within 300 bp below an *r* ^2^ of 0.2 in three of four candidate genes. We identified one SSR and two highly significant SNPs associated with biomass yield. Based on our results, focusing association mapping on candidate gene sequences will be necessary until a dense set of genome-wide markers is available for alfalfa.

**Electronic supplementary material:**

The online version of this article (doi:10.1007/s00122-012-1854-2) contains supplementary material, which is available to authorized users.

## Introduction

Linking DNA polymorphism to trait phenotypic variation is an increasingly important tool for plant breeding programs (Lande and Thompson 1990). Historically, segregating populations of a particular cross have been used to identify marker-trait associations (e.g., Stuber et al. 1999). More recently, association mapping has shown promise for trait mapping due to the increased access to abundant molecular markers in many crops (Stich et al. 2005).

Association mapping takes advantage of the fact that historical recombination within a population has decreased linkage disequilibrium (LD) to short chromosomal intervals, enabling potentially statistically strong and robust marker-trait associations to be detected (Jannink and Walsh 2002). In association mapping, existing allele variation within an entire population can be more efficiently represented because mapping is conducted directly in breeding populations (Hirschhorn and Daly 2005; Remington et al. 2001). In general, the precision of locating a QTL is much higher in association panels compared to biparental mapping populations, provided sufficient markers are available to detect the QTL. If LD extends over long distances, however, the biparental mapping approach is more powerful to detect the existence of a QTL, particularly if marker numbers are limited (Mackay and Powell 2007).

Two major drawbacks exist in association mapping. First, false positive associations between markers and traits can be obtained due to the presence of population structure (Aranzana et al. 2005; Lander and Schork 1994). However, population structure can be assessed with marker information from genome-wide genetic markers (such as SSRs), and association tests can then be conditioned on the population structure to reduce the false positive rate (Aranzana et al. 2005; Pritchard et al. 2000). Second, the extent of LD plays a practical role in determining the number of markers needed to detect associations between genotype and phenotype (Rafalski and Morgante 2004). Limited LD in the population means that associations will only be detected between alleles at loci close together, requiring many markers to saturate the genome (Hagenblad and Nordborg 2002). When the limiting factor for association mapping is the absence of a sufficiently large number of markers evenly dispersed throughout the genome, an alternative strategy is to assay variation in candidate genes (Neale and Savolainen 2004). For both cases, the design and use of association studies require knowledge of the LD structure in the genome (Oraguzie et al. 2007).

Alfalfa is one of the most important forage legumes in the world (Quiros and Bauchan 1988; Michaud et al. 1988), and has been proposed as a bioenergy crop (Delong et al. 1995). Alfalfa has potential to produce high yield but genetic improvement for yield is not as high as has been realized for the major grain crops (Hill et al. 1988). Digestion of forage for animal nutrition or for cellulosic bioethanol production requires the effective hydrolysis of cellulose and solubilization of hemicellulose in the presence of lignin (U.S. DOE 2006). Reducing lignin content can increase the efficiency of sugar release from cell wall complexes up to two fold (Chen and Dixon 2007). Therefore, improving biomass yield and modifying the plant’s cell wall composition are two breeding targets important for both forage and biofeedstock (Ragauskas et al. 2006) applications. If QTL associated with yield and cell wall components could be identified, they could be incorporated into modern cultivars enhancing the efficiency of alfalfa breeding.

Cultivated alfalfa is an autotetraploid (2*n* = 4x = 32) domesticated from the *Medicago sativa*–*falcata* complex. Autotetraploidy complicates genetic mapping, but diploid (2*n* = 2x = 16) relatives of alfalfa exist that share the same karyotype, have highly syntenic genetic linkage groups, and can be hybridized with tetraploid individuals (Diwan et al. 2000; McCoy and Bingham 1988; Quiros and Bauchan 1988). The diploid members of the complex include *M. sativa* subsp. *falcata*, *M. sativa* subsp. *caerulea*, and their natural hybrid, *M. sativa* subsp. *hemicycla* (Quiros and Bauchan 1988; Havananda et al. 2010).

The genomewide extent of LD in the *M. sativa*–*falcata* complex has previously been estimated in one tetraploid breeding population using SSR markers (Li et al. 2011). Within gene LD was estimated in a set of different tetraploid breeding populations using two regions of the alfalfa gene homologous to *M. truncatula* *CONSTANS*-*LIKE* gene (Herrmann et al. 2010). However, both of these populations are expected to have had reduced recombination due to breeding efforts compared to a broad-based natural population. In this paper, we assess both chromosome-wide estimates of LD in a population consisting of 374 unimproved diploid alfalfa genotypes from 120 accessions using 89 polymorphic SSR loci distributed throughout genome and within gene estimates of LD in sequences of four candidate genes of the lignin biosynthesis pathway. In addition, we evaluated SSR and candidate gene SNP marker polymorphisms for associations with 23 traits relevant to biomass accumulation and cell wall components.

## Materials and methods

### Plant materials and phenotyping

We selected 374 individual genotypes from 120 accessions obtained from the USDA National Plant Germplasm System, representing the geographical distribution of the diploid *M. sativa* complex, including subsp. *caerulea*, *falcata*, and *hemicycla* (Supplemental Table 1) (Sakiroglu et al. 2010; Sakiroglu and Brummer 2011). These genotypes were planted in field experiments near Watkinsville and Eatonton, Georgia. The experimental design and procedures were reported previously (Sakiroglu et al. 2011). We evaluated neutral detergent fiber (NDF), acid detergent fiber (ADF) acid detergent lignin (ADL), and total nonstructural carbohydrate (TNC) composition, glucose, xylose, arabinose, total aboveground biomass yield, and regrowth after harvest in 2007 and 2008. Five other agronomic traits were measured in 1 year, stem yield and stem/leaf ratio in 2007, and plant height, stem thickness, and spring regrowth in 2008 (Sakiroglu et al. 2011).

### Genotyping and sequencing

**Fig. 1:**
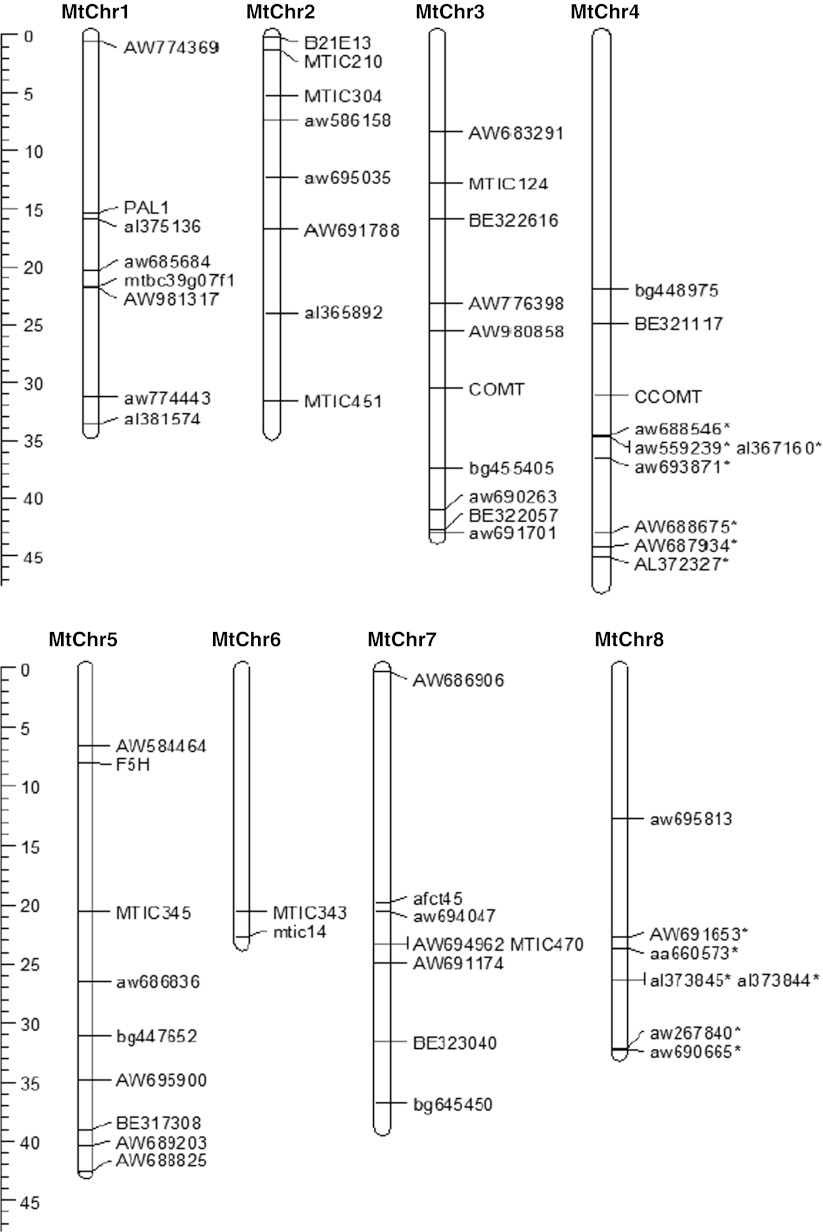
Physical locations of 58 of 89 SSR markers and four candidate genes on *Medicago truncatula* chromosomes, Mt genome sequence version 3.5.1. The ruler indicates scale in Mb

We scored 89 SSR loci on the 374 genotypes and analyzed genetic relationships among them, as described previously (Sakiroglu et al. 2010). The putative physical location of the SSR markers was determined using BLAST to find the sequence of the SSR primers or the EST from which the SSR marker was developed on the genome sequence of *M. truncatula*, version 3.5.1 (www.medicago.org) (Fig. 1).

**Fig. 2:**
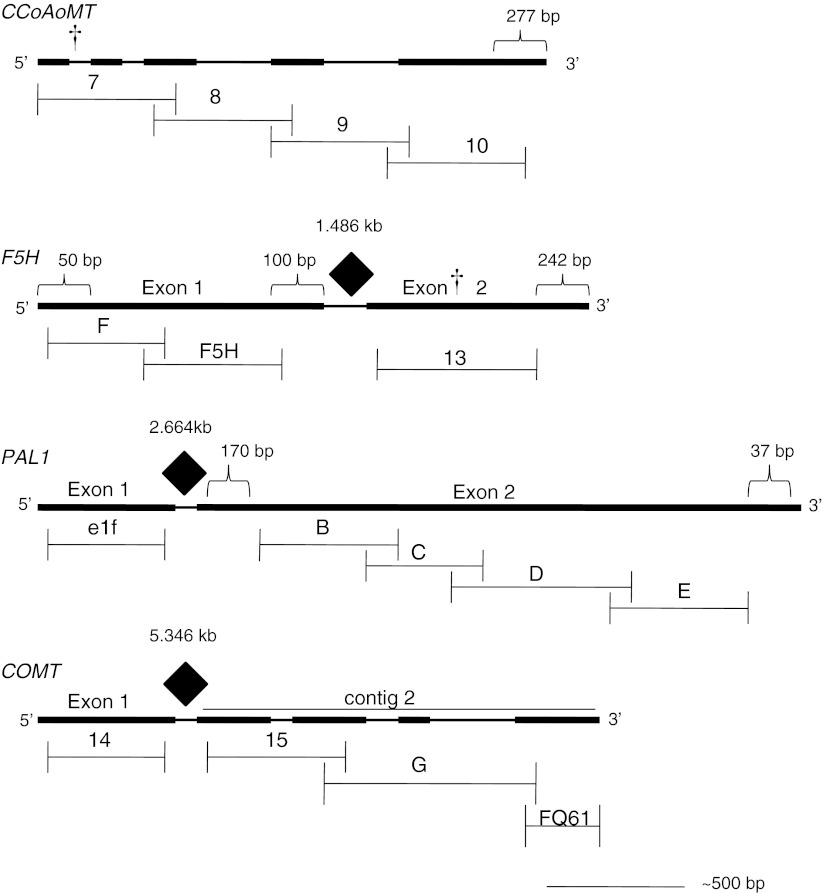
Depiction of candidate genes with overlapping amplicons comprising seven contigs illustrated. Representation of four candidate genes and overlapping amplicons (length of amplicons in bp listed in Supplemental Table 1). Exons are rectangles. *Brackets* indicate the size of unsequenced gene. Three *filled diamonds* indicate introns and sizes determined from *M. truncatula* genome sequence that were not included in the sequencing. *Dagger* indicates location of SNPs significantly associated with biomass yield

We initially targeted to sample one individual from each of 72 accessions from the original 120 accessions for 454 amplicon resequencing (Meyer et al. 2008). However, we later discovered that the accessions denoted as PI 641380 and W6 4794 are a single accession. Therefore, we used 72 individuals from 71 accessions for candidate gene resequencing. We used the subset to ensure adequate sequence coverage of each heterozygous individual in our 454 library. The 71 accessions were selected to represent the subspecies throughout their geographic distribution and SSR marker cluster. We evaluated four genes in the lignin biosynthetic pathway as candidate loci associated with stem composition: *caffeoyl*-*CoA 3*-*O*-*methyltransferase* (*CCoAoMT*), *ferulate*-*5*-*hydroxylase* (*F5H*), *caffeic acid*-*O*-*methyltransferase* (*COMT*), and *phenylalanine ammonialyase* (*PAL 1*). Primers were designed to amplify fragments from these genes ranging in length from 289 to 645 bp. Contiguous fragments were overlapped by 77–327 bp to facilitate haplotype assignments (Supplemental Table 1). Three introns were not amplified presumably due to their excessive length (e.g., *COMT* has an intron greater than 5 kb in *M. truncatula*, which we assume is also large in alfalfa; Fig. 2) and were not included in the 454 library The DNA from 72 individuals was amplified using the Roche Fast Start High Fidelity PCR System (Roche, Branchburg, NJ, USA) following Meyer et al. (2008). Briefly, 30 cycles consisting of a 30 s denaturation step at 95°, 30 s annealing step (temperature listed in Supplemental Table 2) and 30 s extension step at 72°, followed by a final 5 min extension at 72°. Products were visualized on agarose gels. In cases when amplification was weak, with PCR products faint or lacking on agarose gels, PCR products were diluted 1:5 and amplification was repeated using the same PCR conditions. All PCR products were reamplified from amplicons D, E, 15, F, F5H, 8, 9, and 10 (see Fig. 2). A subset of PCR products from amplicons e1f, B, C, 14, G, FQ61, and 13 was weak and therefore was reamplified. No products were reamplified from amplicon 7. Amplicons from each individual were purified using AmPure SPRI beads (Beckman Coulter Genomics, Danvers, MA, USA) and quantified using the Picogreen Assay (Invitrogen, Carlsbad, CA, USA). All amplicons for each individual were pooled in equimolar concentrations and blunt end repaired prior to ligation of individual-specific tags. Tags consisted of eight unique bases, a *Srf1* site and eight complementary bases forming a self annealing hairpin structure (Meyer et al. 2008). The tagged amplicons from each individual were pooled to form a single library. Dephosphorylation and digestion with *Srf1* of tags in the final pool, prior to ligation of library adapters, ensured that only tagged amplicons were sequenced. Roche/454 library preparation and sequencing were done by the Cornell University Life Sciences Core Laboratories Center using Roche *454* Genome Sequencer FLX instruments with Titanium chemistry (Roche Applied Science, Indianapolis, IN, USA) on half of a picotiter plate.

### Management of sequencing reads

Sequences were sorted based on their tags using software provided at http://bioinf.eva.mpg.de/pts/ as described in Meyer et al. (2008), resulting in 72 FASTA files each representing the sequences from a single individual. Sequence reads were assembled for each individual using Lasergene’s SeqMan program (DNASTAR, Madison, WI, USA) to produce seven contigs for each individual (Fig. 2). *M. truncatula* or *M. sativa* sequences used to design the primers for this project were added to each contig as a reference sequence. Each contig was exported from SeqMan as a phrap (.ace) file for use with an in-house Perl script. The script was written to determine single nucleotide polymorphisms (SNPs) and insertion–deletion polymorphisms (indels) within each contig by tallying the number of reads containing the same series of SNPs. Both SeqMan and our Perl script eliminated bases at low frequency (<0.05), variation likely attributable to sequencing errors such as homopolymer regions.

Reamplification of PCR products led to a high rate of chimeric sequences due to recombination during the PCR. As a consequence, our script identified more than two haplotypes for each individual. The script identified the location of each SNP represented by an ambiguity code in the consensus sequence and the number of reads containing each SNP allele at each of those positions. SNP combinations with the highest frequencies, which we presumed were the non-chimeric sequences and replaced the IUPAC ambiguity codes in the consensus sequence to create two likely true haplotypes for each individual for *CCoAoMT* and *F5H*. These manually determined haplotypes from *CCoAoMT* and *F5H* had similar LD plots as the LD analyses based on unphased SNP genotypes. Therefore, we used unphased SNP genotype data to estimate within gene LD as well as to conduct association tests. Manually determined haplotypes had slightly elevated diversity statistics for *CCoAoMT* and slightly lower diversity statistics for *F5H* (Supplemental Table 3) compared to haplotypes determined by PHASE (Stephens and Donnelly 2003), as implemented in DnaSP v. 5.0 (Librado and Rozas 2009). Because the manually determined haplotypes did not show a systematic bias compared to inferred haplotypes based on PHASE, we used the inferred haplotypes for estimating diversity statistics for all genes. Sequences were aligned using MUSCLE (Edgar 2004). Alignments were manually edited using BioEdit (Hall 1999).

### Data analysis

**Fig. 3:**
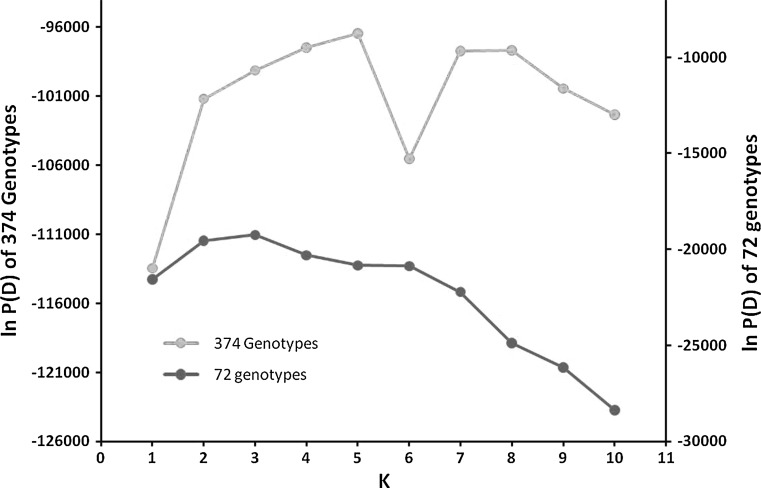
Determining optimal value of *K* using the ad hoc procedure described by Pritchard et al. (2000) for 374 genotypes from 120 accessions (*blue*) and for a sub set of 72 accessions (*red*) (colour figure online)

We used both genome-wide SSRs and candidate gene sequences to test for associations with phenotypic trait data from the field experiment. We inferred population structure using the software program Structure (Pritchard et al. 2000) to analyze the SSR data, as described previously (Sakiroglu et al. 2010). In brief, when all 374 individual genotypes from 120 accessions were considered, the most likely true number of subpopulations (*K*) was five, with each of the groups corresponding to biologically meaningful divisions. The three subspecies clearly separated into distinct clusters, and the subspecies *falcata* and *caerulea* were each further divided into two subgroups. However, among the subset of 72 genotypes selected for candidate gene association, the optimum number of subpopulations (*K*) was found to be three (Fig. 3), each corresponding to one of the subspecies (data not shown). Hence, in the association analyses of SSRs, we used *K* = 5 (Sakiroglu et al. 2010) whereas for association analyses of candidate genes we used *K* = 3. SPAGeDi 1.2 software (Hardy and Vekemans 2002) was used to estimate a kinship matrix for each pair of genotypes (Ritland 1996) using the 89 SSR loci. Negative kinship values were set to zero, following Yu et al. (2006).

**Fig. 4:**
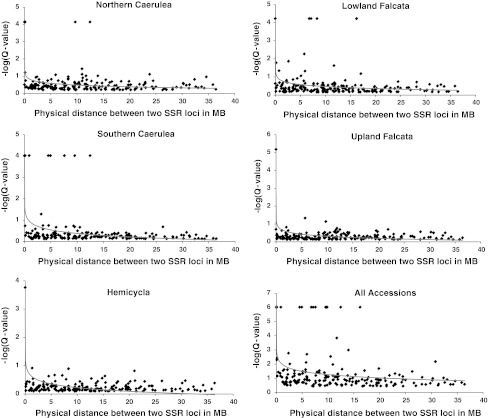
Plots of linkage disequilibrium (−log(*Q* value)) between SSR locus pairs on the same chromosome against their physical distance in Mb, based on the *M. truncatula* genome sequence, in five diploid alfalfa populations and over all 120 accessions

Linkage disequilibrium among SSR markers was determined using the software program GENEPOP 4.0 (Raymond and Rousset 1995), using pairs of loci that were located on the same chromosome based on the *M. truncatula* genome sequence (Fig. 4). The LD between polymorphic sites within the four candidate genes was estimated using TASSEL 3.0 (Bradbury et al. 2007). Because we used a very high number of tests while calculating LD among SSR markers, corrections for multiple testing were performed using the positive false discovery rate (FDR) method (Storey 2002; Storey and Tibshirani 2003) implemented in the software program *Q* Value (Storey 2002).

To estimate genetic diversity in the four candidate genes, we computed the average number of nucleotide differences between sequence pairs, heterozygozity per nucleotide site (π), Tajima’s *D* statistic (Nei 1987; Tajima 1989), and Watterson’s estimator of the population mutation rate (θ) (Watterson, 1975) using the computer program DnaSP v5 (Librado and Rozas 2009). DnaSP does not recognize DNA ambiguity codes. Each individual genotypic sequence resulted in two inferred haplotypic sequences from each individual.

Least square means of 23 phenotypic traits were obtained as described previously (Sakiroglu et al. 2011). The software program TASSEL 2.1 (Bradbury et al. 2007) was used to detect associations between SSR markers and the phenotypic means. TASSEL 3.0 (Bradbury et al. 2007) was used to test for associations between candidate gene SNPs and the phenotypic means. A mixed linear model (MLM) was fitted for each single marker and trait (Yu et al. 2006). In addition to the population structure inference (Q matrix), this approach accounts for relatedness among individuals using the pairwise kinship matrix as a covariate in the mixed model. Correction for multiple testing was applied to *P* values obtained from MLM using the positive FDR method (Storey 2002; Storey and Tibshirani 2003) implemented in software program *Q* Value (Storey 2002). We also constructed quantile–quantile (QQ) plots to visualize the observed MLM *P* value versus expected *P* value distribution for each of the candidate gene association tests. Deviations from the line of equality imply an association.

Alignments for each gene region have been deposited at GenBank with the following accession numbers. F5H exon 1 JN705257–JN705321; F5H exon 2 JN714201–JN714257; PAL 1 exon 1 JN849691–JN849757; PAL 1 exon 2 JN849758–JN849828; COMT exon 1 JN849829–JN849897; COMT contig 2 JN849970–JN850038; CCoAoMT JN849898–JN849969.

## Results

### Sequencing results and molecular diversity of subspecies

A total of 370,779 reads with an average length of 453 bp was generated from the tagged amplicon library of four candidate genes. One hundred and sixteen of 1,152 total amplicons were missing or lacked sufficient coverage for further analysis. Thus, the range of read coverage for each contig was from 0 to over 2,000 (Table 1). In general missing amplicons were not clustered within one individual, however, there were three exceptions; one individual from subspecies *caerulea* (PI 631922) and one from subspecies *hemicycla* (PI 631814) were missing eight of the 16 amplicons while one individual from subspecies *falcata* (PI 577558) was missing seven of 16 amplicons. *COMT* contig 2 had the highest level of missing data. Three individuals lacked suitable read coverage for *COMT* contig 2 to be included in any analyses and 27 individuals were missing one of the three amplicons.

**Table 1:** Candidate gene amplicon composition, coverage and SNP (MAF > 0.05) distribution

Contig	Number of amplicons	Alignment length (bp)	Coverage range (reads)	No. of SNPs	No. of SNPs per bp	No. of NS SNPs	No. of indels
*CCoAoMT*	4	1,185	0–1,886	31	1:38	3	9
*F5H* exon 1	2	723	0–1,499	16	1:45	3	0
*F5H* exon 2	1	594	0–287	39	1:15	5	0
*PAL1* exon 1	1	377	0–207	14	1:30	1	0
*PAL1* exon 2	4	1,481	0–2,014	52	1:28	0	0
*COMT* exon 1	1	415	0–1,182	11	1:38	2	0
*COMT* contig 2	3	1,224	0–1,536	31	1:39	0	1

Contigs are illustrated in Fig. 2

**Fig. 5:**
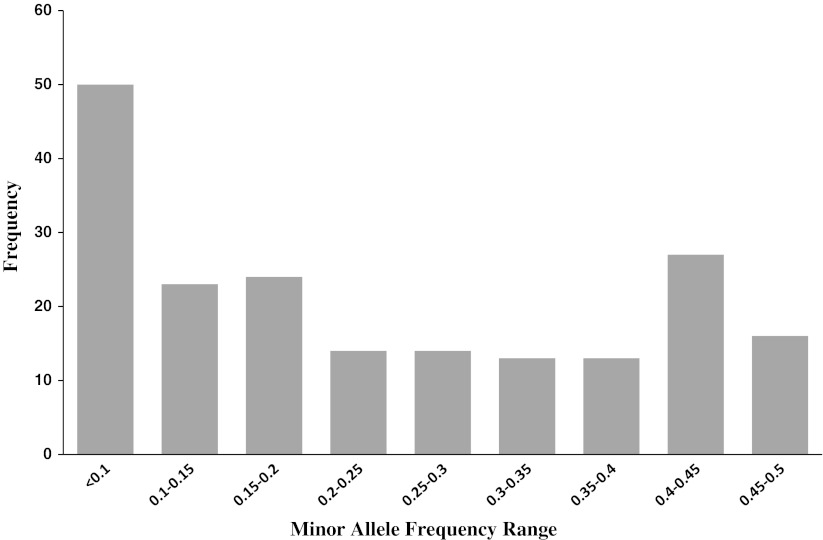
Minor allele frequencies of 194 SNP discovered in seven contigs of the four candidate genes sequenced

Alignments of genotype sequences across the seven contigs yielded 194 SNPs with minimum allele frequencies (MAF) above 0.05 (Table 1, Fig. 5). *F5H* exon 2 had the greatest number of SNPs per bp (1 SNP for every 15 bp) as well as the highest number of SNPs resulting in nonsynonymous substitutions (5 SNPs). *CCoAoMT* and *COMT* contig 2 are the only contigs that include introns and indels. *CCoAoMT* had nine indels. *COMT* contig 2 had only one indel.

We estimated molecular genetic diversity parameters from the inferred haplotype sequences of the four candidate genes. Overall measures of the expected heterozygosity per nucleotide site (π) ranged from 0.008 (*PAL1* exon 2) to 0.0257 (*F5H* exon 2) with an average of 0.0122. The number of polymorphic sites per gene corrected for sample size, θ, ranged from 0.0097 (*Pal 1* exon 2) to 0.0197 (*F5H* exon 2) with an average of 0.0142 (Table 2). Since θ estimates roughly correspond to heterozygosity, our results suggest that the gene sequences from two randomly chosen wild alfalfa accessions would differ on average once every 71 bases (i.e., 1/0.0142). *F5H* exon 2 had the highest number of polymorphic sites, despite being one of the shortest contigs and therefore was the most diverse contig based on both of the molecular diversity parameters evaluated here. Tajima’s test of neutrality was significant in *F5H* exon 2 for subspecies *hemicycla*. Despite having low sequence diversity in *COMT* contig 2, Tajima’s test of neutrality was also significant for *COMT* contig 2 in subspecies *falcata* (Table 2). Negative values of *D* indicate an excess of rare alleles.

**Table 2:** Summary of DNA sequence variation from four candidate genes in three subspecies of diploid alfalfa from inferred haplotypes

*Medicago sativa* subspecies	No. of individuals	No. of polymorphic sites	π	θ	Tajima’s *D*	No. of haplotypes	Haplotype diversity (SD)
*CCoAoMT* (1,340 bp)							
*caerulea*	26	14	0.0122	0.0115	0.176	22	0.911 (0.025)
*falcata*	36	20	0.0127	0.0148	−0.438	25	0.844 (0.026)
*hemicycla*	10	11	0.0127	0.0117	0.331	10	0.926 (0.032)
Overall	72	21	0.0116	0.0158	−0.750	42	0.915 (0.012)
*F5H* exon 1 (723 bp)							
*caerulea*	23	21	0.0056	0.0068	−0.491	19	0.804 (0.058)
*falcata*	33	39	0.008	0.012	−1.069	32	0.909 (0.027)
*hemicycla*	9	16	0.0065	0.0064	0.004	6	0.850 (0.006)
Overall	65	52	0.0095	0.0137	−0.956	54	0.926 (0.015)
*F5H* exon 2 (594 bp)							
*caerulea*	21	42	0.0141	0.017	−0.641	16	0.785 (0.063)
*falcata*	28	52	0.0201	0.0202	0.005	30	0.901 (0.034)
*hemicycla*	8	30	0.0085	0.0162	−1.981^a^	6	0.617 (0.135)
Overall	57	57	0.0257	0.0197	0.971	47	0.904 (0.020)
*PAL1* exon 1 (377 bp)							
*caerulea*	25	24	0.0143	0.0146	−0.129	16	0.847 (0.38)
*falcata*	33	19	0.0109	0.0123	−0.350	26	0.943 (0.012)
*hemicycla*	9	14	0.0114	0.0116	0.058	12	0.922 (0.051)
Overall	67	33	0.0143	0.018	−0.620	43	0.945 (0.008)
*PAL1* exon 2 (1,483 bp)							
*caerulea*	25	21	0.0048	0.007	−1.035	19	0.780 (0.06)
*falcata*	35	38	0.0121	0.0108	0.405	54	0.991 (0.004)
*hemicycla*	9	36	0.0069	0.011	−1.686	13	0.954 (0.034)
Overall	69	22	0.0075	0.0097	−0.645	37	0.853 (0.025)
*COMT* exon 1 (415 bp)							
*caerulea*	26	15	0.0082	0.0085	−0.136	11	0.732 (0.056)
*falcata*	34	21	0.0101	0.0111	−0.285	19	0.802 (0.046)
*hemicycla*	9	11	0.0066	0.008	−0.801	8	0.83 (0.064)
Overall	69	26	0.0094	0.0118	−0.597	29	0.870 (0.018)
*COMT* contig 2 (1,203 bp)							
*caerulea*	14	29	0.0068	0.0063	0.254	18	0.918 (0.044)
*falcata*	23	45	0.0038	0.0089	−2.123^a^	22	0.84 (0.051)
*hemicycla*	5	44	0.0121	0.0143	−0.741	9	0.978(0.054)
Overall	42	62	0.0071	0.0108	−1.336	45	0.933 (0.019)
Average Overall			0.0122	0.0142			

^a^Tajima’s *D* values significantly deviated from zero (p < 0.05)

### Linkage disequilibria

Physical locations of 58 of 89 SSR loci were identified using the *M. truncatula* genome sequence build (version 3.5.1), which covers about 66 % of the gene space (Chris Town, pers. comm.). We could estimate LD from 199 locus pairs between markers known to be located on the same chromosome (Fig. 1). Markers on *M. truncatula* chromosomes 4 and 8 that are denoted in Fig. 1 by asterisks are most likely found on the other chromosome in *M. sativa*, because the sequenced *M. truncatula* accession has an unusual translocation between chromosomes 4 and 8 (Kamphuis et al. 2007). To investigate the evidence of the translocation in depth, we calculated LD among SSR markers that are denoted in Fig. 1 by asterisks with remaining markers of both chromosomes 4 and 8 separately. We observed only two significant associations when all accessions were considered: SSR marker BE321117 showed significant LD with al367160 on chromosome 4 and with aw267840 on chromosome 8 (Fig. 1). However, when the five groups identified by Structure were analyzed separately, no significant LD was detected, suggesting the observed LD was created by family structure rather than a real physical proximity. We excluded a total of 20 pairwise LD calculations because we could not infer the accurate distance between markers in the above-mentioned situation.

The average pairwise distance between the 179 SSR marker pairs was 11.9 Mbp. We found little LD among SSR markers. To allow better visualization, we converted *P* values of marker pairs that showed LD to −*log* (*P* *value*) (Fig. 4). If we consider *P* values of 0.0001 or lower [i.e., a −*log* (*P* *value*) ≥4] to indicate true LD, then we conclude that, in general, LD decays very quickly. Disregarding the five groups previously detected (Sakiroglu et al. 2010), 15 cases of LD (8.4 %) extending as long as 20 Mb were observed—although at least some of these were probably due to population structure (Fig. 4). When the five groups were analyzed separately, the number of SSR locus pairs in LD in each group was reduced compared to the overall number of associations. Only one SSR locus pair was in LD in *hemicycla*, corresponding to 0.56 %, possibly a function of small population size. Although upland *falcata* and southern *caerulea* had the same number of individuals, the number of SSR locus pairs in LD was five times higher in southern *caerulea* (5.6 %) compared to upland *falcata* (1.1 %) (Table 3).

**Table 3:** Number of SSR locus pairs showing linkage disequilibrium in five main populations of diploid alfalfa and over all genotypes based on a significance level of *P* = 0.0001 after control for the false discovery rate (FDR)

Groups	No. of genotypes	No. of locus pairs in LD	% of locus pairs in LD
Southern caerulea	99	10	5.6
Northern caerulea	69	5	2.8
Hemicycla	44	1	0.56
Lowland falcata	63	6	1.3
Upland falcata	99	2	1.1
Overall	374	15	1.8

**Fig. 6:**
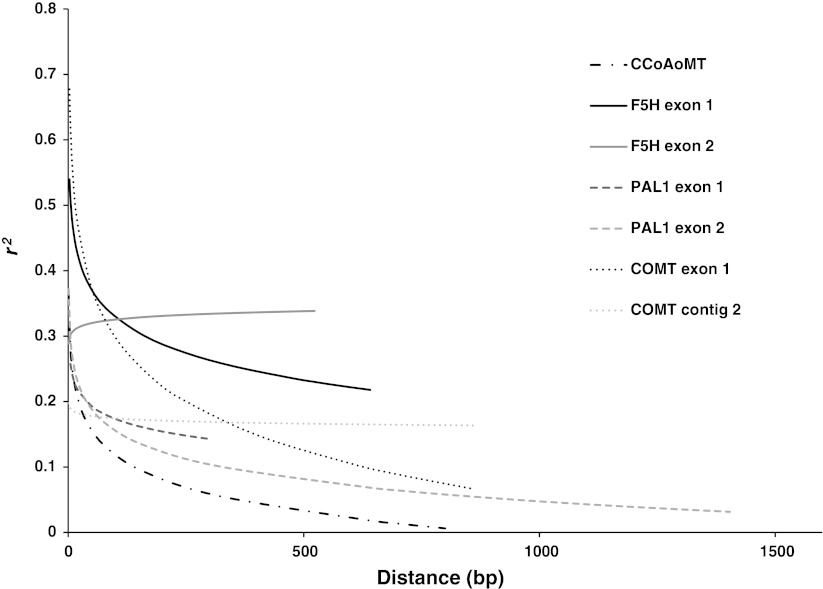
LD plots of squared correlations of allele frequencies (*r* ^2^) against distance between polymorphic sites for four candidate genes in seven contigs as depicted in Fig. 2

The extent of LD varied among the candidate gene sequences (Fig. 6). Linkage disequilibrium decayed below *r* ^2^ = 0.1 within 750 bp in three of the seven contigs. In *CCoAoMT* and *PAL 1* exon 2, LD decayed rapidly to below *r* ^2^ = 0.1 within 500 bp. In *PAL 1* exon 1, LD decayed to an *r* ^2^ = 0.14 in the 377 bp length of the sequence. For *F5H* exon 1, LD did not decay below *r* ^2^ = 0.2 within the 723 bp length, although the trend showed a decrease in LD with increased distance. *COMT* contig 2 had an average *r* ^2^ = 0.17 and LD did not decay across the 1,203 bp region. LD was highest in *F5H* exon 2 with an average *r* ^2^ = 0.33 and did not decay. The pattern of linkage disequilibrium in *F5H* exon 2 for each subspecies indicates that linkage disequilibrium was strongest in subspecies *hemicycla* (Supplementary Figure 1). *COMT* contig 2 and *F5H* exon 2 had significant values for Tajima’s *D* in subspecies *falcata* and *hemicycla*, respectively (Table 2).

### Tests of association

Based on genomewide association analysis with 89 SSR loci and 23 phenotypic traits, we found only one weak association (FDR *Q* value = 0.037) with total biomass yield in 2007 (Table 4). The SSR marker *mtic14* is located on Chromosome 6 according to the *M. truncatula* genome sequence build 3.5; however, it had previously been mapped to linkage group 2 in alfalfa (Robins et al. 2007). The marker did not show any LD with the other SSR loci known to be located on chromosomes 2 or 6.

**Table 4:** Significant marker-phenotype associations after correction for multiple testing using the positive FDR method. (SNP FDR *Q* values <0.05)

Trait	Year	Marker/position	Linkage group	*F*	Marker effect		FDR *Q* value
					*P*	*r* ^2^	
Yield	2007	*CCOAOMT/*111	4	21.96	8.50E−08	8.50E−08	5.64E−05^a^
Stem proportion	2007	*CCOAOMT/*111	4	15.01	5.78E−06	5.78E−06	0.0013
Yield	2008	*CCOAOMT/*111	4	17.77	1.01E−06	1.01E−06	0.0003
Stem thickness	2008	*CCOAOMT/*111	4	8.91	4.31E−04	4.31E−04	0.0476
Regrowth	2007	*CCOAOMT/*1006	4	9.65	2.32E−04	2.32E−04	0.0308
Yield	2008	*F5H* exon 2/276	5	10.74	1.55E−05	0.5682	0.0094^a^
Yield	2008	SSR, MTIC14	6	2.8	4.71E−05		0.037

^a^Significant associations supported by QQ plots

**Fig. 7:**
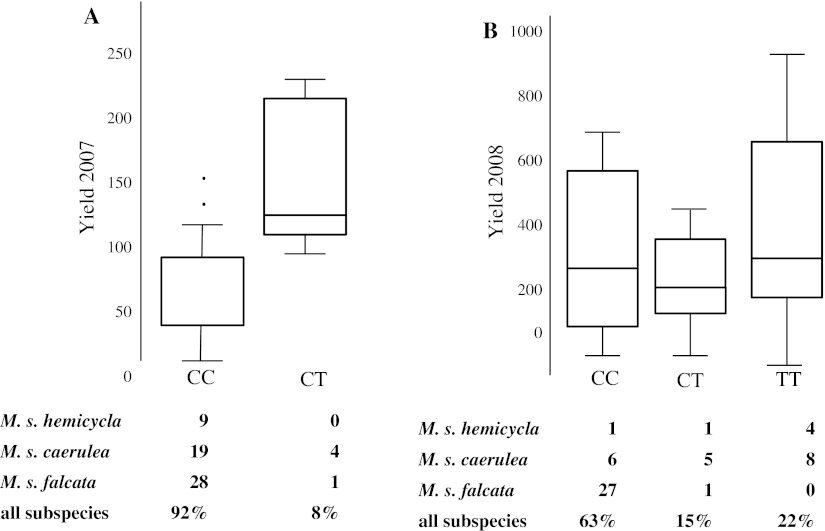
Significant SNP-trait associations (**a**) *CCoAoMT* SNP 111 and yield 2007. Phenotypic effects of genotypes at position 111. Percentage of individuals sampled with each genotypic class for each subspecies and overall subspecies. **b** Significant SNP-trait association between *F5H* exon 2 SNP 276 and yield 2008. Phenotypic effects of genotypes at position 276. Percentage of individuals sampled with each genotypic class for each subspecies and overall subspecies

Tests of association between 194 SNPs with a minimum allele frequency (MAF) >0.05 from the four candidate genes with 23 phenotypic traits resulted in seven significant associations with FDR *Q* values <0.05 (Table 4). Additional SNP-trait associations with *P* values <0.05 but that were not significant after correcting for multiple testing are listed in Supplemental Table 4. Only two SNP-phenotype associations were identified when quantile–quantile (QQ) graphs were plotted (Supplementary Figure 2). One marker, SNP 111 in *CCoAoMT*, was associated with total biomass yield in both 2007 and 2008, stem proportion in 2007, and stem thickness in 2008. *CCoAoMT* SNP 111 is located in the first intron (Fig. 2) and not linked to any other SNPs. Increased yield in 2007 was associated with the C/T genotype at position 111. Only five individuals of 61 (8 %) for which we had sufficient sequence coverage had a thymine at that position, including four *M. sativa* subsp. *caerulea* and one *M. sativa* subsp. *falcata* genotypes (Fig. 7a). A SNP in *F5H* exon 2 was associated with yield in 2008. The SNP at position 276 is a synonymous substitution (Fig. 2). The highest yield values were associated with the homozygous TT condition found in ten of 56 individuals (Fig. 7b). Another SNP in *CCoAoMT* was associated with regrowth in 2007.

## Discussion

### Candidate gene sequencing and sequence diversity

For sequencing in this project, we multiplexed four genes consisting of seven contigs derived from 16 overlapping tagged amplicons and aimed for 600× coverage using Titanium chemistry on the 454 FLX sequencer. Our amplicon lengths were approximately 500 bp, but we were not certain about the read lengths we could expect from the Titanium chemistry when we started the experiment. We were also concerned that our estimation of the concentrations of the 1,200 amplicon reactions was accurate. Taken together, this uncertainty supported our conservative approach. Our experience from this study indicates that accurate haplotype deduction can be achieved with as few as 35 454 FLX reads because, at this coverage level, sequencing errors can still be easily distinguished from true SNPs. The advantages of 454 sequencing over Sanger and Illumina methods are that read lengths allow for the determination of phase over longer distances and insertion-deletion mutations can be easily deduced, both of which are useful for distinguishing haplotypes. Template concentration for PCR, amplicon quantification for pooling prior to sequencing, and methods to avoid PCR recombination which we encountered and that has been seen by other groups (Griffin et al. 2011) are all needed to ensure even coverage of 35–100X.

A previous study investigating the history of domestication of alfalfa (Muller et al. 2005) reported higher levels of sequence diversity for diploid *M. sativa* subsp. *caerulea* than we report here. Sequence diversity at two genes sampled from eight individuals resulted in θ = 0.0376 and θ = 0.0272 while our values ranged from 0.0048 to 0.0143. The strategy used by Muller et al. (2005) was very different from ours in a few ways. First, only one allele per individual was used in an effort to sample more variation across a diverse collection of *Medicago*. Second, the plant material used in their study included diploid and tetraploid, domesticated, and wild populations. Finally, their sequences were from predominantly intron regions.

Comparing patterns of genetic diversity across species could be hampered due to differences in the mode of reproduction (self pollinated vs. cross pollinated) and the nature of genetic material used (breeding material vs. unimproved population). Alfalfa has a predominantly outcrossing breeding system and the plant material used in this experiment is unimproved germplasm collected from broad-based populations. Although we observed higher sequence diversity in the four candidate genes compared to other crop species and model plants (Tenaillon et al. 2001; Schmid et al. 2005; Liu and Burke 2006; Mather et al. 2007), we focused on comparing our results to the wild relatives or landraces of maize (*Zea mays* ssp. *mays* L.) and sunflower (*Helianthus annuus* L.), which are also outcrossing populations and could be assumed to be more similar to alfalfa than populations from autogamous species. The average θ value estimated in this study (0.0142) was comparable to the values from 21 genes in 15 maize landraces (θ = 0.0129; Tenaillon et al. 2001) and from nine genes in 16 wild populations of sunflower (θ = 0.0144; Liu and Burke 2006).

Tajima’s test of neutrality indicated that selection may be acting on *F5H* exon 2 within subspecies *hemicycla*, but not in the other subspecies. Sliding window analysis of *F5H* exon 2 did not detect significant values for Tajima’s *D* within subspecies *caerulea* or *falcata*, but Tajima’s *D* was consistently negative for *hemicycla*. Negative Tajima’s *D* values indicate an excess of rare variation, which may be due to *hemicycla*’s hybrid nature, consisting of genetic variation derived from both of the other subspecies. Tajima’s test of neutrality is negative for *COMT* contig 2 in subspecies *falcata*, suggesting that selection has acted on this sequence.

### Linkage disequilibria

The extent of LD is crucial to determine marker density necessary for association mapping analyses, with longer LD requiring fewer markers to saturate the genome, but resulting in lower resolution (Jorde 1995; Buckler and Thornsberry 2002; Ching et al. 2002; Rafalski and Morgante 2004). We observed very little LD between SSR marker pairs and the estimates of the extent of LD in our study are lower than those reported in maize and barley (Remington et al. 2001; Liu et al. 2003; Stich et al. 2005; Malysheva-Otto et al. 2006). The small number of SSR locus pairs in LD could partially be due to the FDR calculations that we used to correct possible false positives arising from thousands of pairwise LD calculations. It could also be due to the nature of the plant material. In the above-mentioned studies, landraces or inbred lines that had resulted from human selection were used, which could create LD (Jannink and Walsh 2002), whereas our germplasm contained all wild accessions. The extent of LD in alfalfa was previously estimated in a breeding population using SSR markers and the results revealed that 61.5 % of SSR marker pairs separated by less than 1 Mbp were in LD (*P* < 0.001) implying extensive LD (Li et al. 2011). However, Li et al. (2011) used a synthetic tetraploid alfalfa population that was derived from 300 individuals of three cultivars (100 individuals from each cultivar). The larger estimate of the extent of LD obtained by Li et al. (2011) compared to LD in our study was probably due to artificial selection.

LD is considered to have decayed when *r* ^2^ values drop below 0.1 (Remington et al. 2001, Ersoz et al. 2007). Two of our gene sequences decayed below *r* ^2^ = 0.1 in 500 bp and overall LD was below 0.2 within 500 bp in five of the seven contigs. Only *F5H* exon 2 compares to a previous report of within gene LD in alfalfa. Estimates of within gene LD in a *CONSTANS*-*LIKE* gene from 59 genotypes of a breeding variety in tetraploid alfalfa revealed that LD of *r* ^2^ = 0.2 could persist as long as 700 bp (Herrmann et al. 2010). The difference between the LD estimates in two studies is probably attributed to usage of different genetic material. Herrmann et al. (2010) used cultivated material in which LD could persist over longer distances due to bottlenecks produced by artificial selection (Ching et al. 2002; Liu and Burke 2006; Kolkman et al. 2007), where as we used broad-based wild germplasm. The difference in the extent of LD between different genetic materials has previously been reported in other crops (Caldwell et al. 2006; Liu and Burke 2006).

### Association analyses

We identified one SSR marker, and three SNPs associated with biomass yield, stem proportions, regrowth, and stem thickness but no associations with cell wall composition traits. Given the relative paucity of SSR markers we examined and the limited number of individuals (and few genes) in our candidate gene analysis, this lack of association is not surprising. The *CCoAoMT* SNP in position of 111 associated with several traits is located in the first intron and although it is not in linkage disequilibrium with any SNPs in the first exon or downstream of this site, it may be linked to causative SNPs in the promoter region. The *F5H* exon 2 SNP associated with yield in 2008 is a synonymous change; however, LD does not decay within the region we sequenced, so this SNP may be linked functionally to an unsampled causative SNP. The nature of each of these associations needs to be investigated further and validated in additional alfalfa populations.

Successful candidate gene association mapping studies have generally focused on genes from single pathways (Myles et al. 2009). Despite evidence that the lignin biosynthetic genes *CCoAoMT* and *F5H* directly impact the lignin content of alfalfa (Guo et al. 2001; Chen and Dixon 2007), our candidate gene approach did not detect any associations with the cell wall characteristics measured, which were based on fiber analysis. Weak associations between lignin genes and both yield traits and cell wall components have been reported previously from maize inbred lines by Chen et al. (2010), who concluded that qualitative trait polymorphisms for yield and cell wall characteristics segregate independently of one another. The phenylpropanoid pathway, of which lignin is one of many products, has several components that have been linked to plant growth.

In summary, in this paper we attempt to estimate both genome-wide SSR and within gene SNP variation to determine the extent of LD in diploid alfalfa. In terms of the potential to use the candidate gene approach for allele mining for alfalfa improvement, we have shown that although our sample size was small, two significant SNPs in two candidate genes that are associated with biomass yield and other traits were detected.

## Electronic supplementary material

Below is the link to the electronic supplementary material.

Supplementary material 1 (PDF 495 kb)
