# Supplementary material for: Patterns of linkage disequilibrium and association mapping in diploid alfalfa (M. sativa L.)
Source: Theor Appl Genet. 2012 Apr 5;125(3):577–90. doi: 10.1007/s00122-012-1854-2 (PMC3397135; doi:10.1007/s00122-012-1854-2)

Patterns of Linkage Disequilibrium and Association Mapping in Diploid Alfalfa (*M. sativa* L.)

Muhammet Sakiroglu, Sue Sherman-Broyles, Alec Story, Kenneth J. Moore, Jeffery J. Doyle, and E. Charles Brummer

Muhammet Sakiroglu

Department of Biology, Kafkas University, Kars, Turkey, 36100

Sue Sherman-Broyles

Dep. of Plant Biology Cornell Univ. Ithaca, NY 14853

Alec Story

Dep. of Plant Biology, Cornell Univ. Ithaca, NY 14853

Kenneth J. Moore

Dep. of Agronomy, Iowa State Univ., Ames, IA 50011

Jeffery J. Doyle

Dep. of Plant Biology, Cornell Univ. Ithaca, NY 14853

E. Charles Brummer (Corresponding Author)

Samuel Roberts Noble Foundation,

2510 Sam Noble Parkway,

Ardmore, OK 73401

ecbrummer@noble.org

Phone: (1)580 224 6804

Fax: (1) 580 224 6802

Supplemental Table 1. List of all the accessions used in this study along with the classification of each accession, country of origin, number of individual genotypes used in this study.

PI Number	Classification	Number of Genotypes Used	Country of Origin
PI 179370[a]	Southern caerulea	4	Turkey
PI 210367[a]	Southern caerulea	4	Iran
PI 212798[a]	Southern caerulea	4	Iran
PI 222198[a]	Upland falcata	1	Afghanistan
PI 243225	Southern caerulea	4	Iran
PI 251690[a]	Upland falcata	1	Former Soviet Union
PI 251830[a]	Lowland falcata	3	Austria
PI 258752[a]	Upland falcata	2	Russia
PI 283640	Southern caerulea	4	Former Soviet Union
PI 299045[a]	Northern caerulea	4	Russian Federation
PI 299046	Northern caerulea	4	Russian Federation
PI 307395[a]	Northern caerulea	4	Former Soviet Union
PI 314267[a]	Southern caerulea	4	Uzbekistan
PI 314275	Southern caerulea	4	Uzbekistan
PI 315460	hemicycla	2	Russian Federation
PI 315462[a]	Northern caerulea	1	Russian Federation
PI 315466	Northern caerulea	4	Russian Federation
PI 315480	Upland falcata	4	Russia
PI 325387[a]	Upland falcata	2	Russia
PI 325396[a]	Upland falcata	3	Russia
PI 325399[a]	Upland falcata	3	Russia
PI 440500	Southern caerulea	4	Kazakhstan
PI 440501	Southern caerulea	4	Kazakhstan
PI 440502	Southern caerulea	4	Kazakhstan
PI 440505	Southern caerulea	4	Kazakhstan
PI 440507[a]	Southern caerulea	4	Kazakhstan
PI 440514	Southern caerulea	2	Kazakhstan
PI 464712	Southern caerulea	4	Turkey
PI 464713	Southern caerulea	4	Turkey
PI 464714[a]	Southern caerulea	3	Turkey
PI 464715[a]	Southern caerulea	4	Turkey
PI 464717	Southern caerulea	3	Turkey
PI 464718	Southern caerulea	4	Turkey
PI 464719	Southern caerulea	4	Turkey
PI 464720	Southern caerulea	1	Turkey
PI 464721	Southern caerulea	4	Turkey
PI 464722[a]	Southern caerulea	4	Turkey
PI 464723[a]	Southern caerulea	2	Turkey
PI 464724	Southern caerulea	3	Turkey
PI 464726[a]	Southern caerulea	3	Turkey
PI 464727	hemicycla	2	Turkey
PI 464728[a]	hemicycla	3	Turkey
PI 464729	Upland falcata	2	Turkey
PI 486205	Upland falcata	3	Russia
PI 486206[a]	Upland falcata	3	Russia
PI 486207	Upland falcata	4	Russia
PI 494662[a]	Lowland falcata	4	Romania
PI 502425[a]	Northern caerulea	1	Russia
PI 502437	Northern caerulea	3	Russian Federation
PI 502438	Upland falcata	1	Russia
PI 502447[a]	Lowland falcata	3	Russia

PI 502448 [a]	Lowland falcata	4	Russia
PI 502449 [a]	Lowland falcata	4	Former Soviet Union
PI 505871 [a]	Northern caerulea	3	Former Soviet Union
PI 538987 [a]	Lowland falcata	2	Russia
PI 577541 [a]	Northern caerulea	3	Kazakhstan
PI 577543 [a]	hemicycla	4	Georgia
PI 577545	Northern caerulea	1	Russian Federation
PI 577546 [a]	Northern caerulea	4	Georgia
PI 577547	Northern caerulea	4	Georgia
PI 577548 [a]	Northern caerulea	4	Russian Federation
PI 577549	Southern caerulea	2	Georgia
PI 577551 [a]	Northern caerulea	4	Canada
PI 577552	Northern caerulea	2	Canada
PI 577555 [a]	Upland falcata	4	Ukraine
PI 577556 [a]	Upland falcata	4	Bulgaria
PI 577558 [a]	Lowland falcata	3	Russia
PI 577564 [a]	Lowland falcata	2	Russia
PI 631546	Upland falcata	3	Russia
PI 631549	Upland falcata	2	Russia
PI 631556 [a]	Upland falcata	1	Russia
PI 631561 [a]	Lowland falcata	4	Switzerland
PI 631566 [a]	Lowland falcata	2	Bulgaria
PI 631568 [a]	Lowland falcata	4	Italy
PI 631571 [a]	Lowland falcata	2	Bulgaria
PI 631577 [a]	Lowland falcata	2	Italy
PI 631650 [a]	Lowland falcata	4	Bulgaria
PI 631652	Upland falcata	4	Russia
PI 631654 [a]	Upland falcata	3	Russia
PI 631656 [a]	Upland falcata	3	Russia
PI 631658 [a]	Upland falcata	3	Russia
PI 631660	Upland falcata	4	Russia
PI 631661	Upland falcata	3	Russia
PI 631666 [a]	Upland falcata	4	Russia
PI 631667	Upland falcata	4	Russia
PI 631668	Upland falcata	4	Russia
PI 631689 [a]	Lowland falcata	3	Bulgaria
PI 631691 [a]	Lowland falcata	2	Bulgaria
PI 631707 [a]	Upland falcata	1	China
PI 631807 [a]	Lowland falcata	3	Russia
PI 631808	Lowland falcata	1	Russia
PI 631809	Lowland falcata	2	Russia
PI 631812	Upland falcata	3	Russia
PI 631813	Lowland falcata	4	Russia
PI 631814 [a]	hemicycla	3	Russia
PI 631816	Upland falcata	4	Russia
PI 631817	Upland falcata	2	Russia
PI 631818 [a]	Lowland falcata	3	Russia
PI 631829 [a]	Upland falcata	3	Russia
PI 631842	Lowland falcata	1	Sweden
PI 631921 [a]	Northern caerulea	4	Russian Federation
PI 631922 [a]	Northern caerulea	4	Kazakhstan
PI 631924 [a]	Southern caerulea	4	Armenia
PI 631925 [a]	Northern caerulea	4	Kazakhstan
PI 631926 [a]	Northern caerulea	4	Russian Federation
PI 634034	Upland falcata	1	Russia
PI 634106 [a]	Upland falcata	2	Ukraine

PI 634119 [a]	hemicycla	4	Kazakhstan
PI 634136 [a]	hemicycla	4	Kazakhstan
PI 634174 [a]	hemicycla	4	Kazakhstan
PI 634176 [a]	hemicycla	3	Kazakhstan
PI 641380 [a]	Northern caerulea	7	Russian Federation
PI 641543	Upland falcata	4	Mongolia
PI 641544	Upland falcata	4	Mongolia
PI 641601	hemicycla	4	Kazakhstan
PI 641603 [a]	hemicycla	3	Kazakhstan
PI 641606	hemicycla	3	Kazakhstan
PI 641615 [a]	hemicycla	4	Kazakhstan
PI 641619 [a]	hemicycla	1	Kazakhstan
SD201 [a]	Upland falcata	1	

[a] Accessions selected for candidate gene association study

Supplemental Table 2. Primers used for PCR, sequence and annealing temperatures. Approximate size of products and estimated size of sequence overlap between contiguous sequences.

Amplicon	Primer Name	primer sequence	annealing temp	product length	overlap 5'	overlap 3'
Pal 1						
e1f	PalExon1F	atggagggaattaccaatggc	58	377	NA	NA
	PalExon1R	ttctgcaaggcaccaccttgttt				
B	Pal1aF	atattaccccatgtttaccacttc	55	490	NA	216
	Pal1aR	acgatcttgtttaggcttttg				
C	Pal1bF	agcggttttgtctgaagttatgtc	56	435	216	194
	Pal1bR	gagtttaccgatcgaagcaagag				
D	Pal1cF	atggcttggtcctttgattga	62	557	194	101
	Pal1cR	cttggcttacagtgttcttgac				
E	Pal1dF	actctttgggtttgatttcttcta	55	403	101	NA
	Pal1dR	tcctggcgatatcac				
Comt						
14	454ComtF4	ggttcaacaggtgaaact	52	416	NA	NA
	454ComtR418	agctttccatgagcactt				
15	454ComtF5769	accacctaaaagatgcag	53	514	NA	76
	454ComtR6282	cagcatcagcctttggaa				
G	ComtcF	atttgatgttttgtgtaggagttg	59	629	76	88
	ComtcR	ttgtggccaggcttgaatct				
FQ61	ComtFQ61F	gctatgaagcactaccagacaatgg	58	289	88	NA
	ComtFQ61R	gcaacacgccaaagaacttaaacc				
F5H						
F	F5HaF	tcacgaatcctcaaaagacc	55	571	NA	301
	F5HaR	gaaatattaaaagctccaaacaa				
F5H	F5H_F	gacatggcgttcgctcactac	58	456	301	NA

	F5H_R	gacatggcgttcgctcactac				
13	454F5HF2369	cgtgatgtttggaggaac	54	612	NA	NA
	454F5HR2981	ggacacaagacacgctta				
CCoAoMT						
7	454CcomtF46	tggcaaccaacgaagatcaaa	55	507	NA	259
	454CcomtR500	tagacaccaatttccatggt				
8	454CcomtF296	cacagcaaaacacccatg	TD 55-50	540	259	254
	454CcomtR786	aattacaggtagacccaa				
9	454CcomtF580	attcctgaagatggaaag	TD 55-50	645	254	327
	454CcomtR1079	ccactttaacaagatcaa				
10	454CcomtF769	ttgggtctacctgtaatt	TD 55-50	535	327	NA
	454CcomtR1272	ttgatcctacggcagata				

Supplemental Table 3. Diversity statistics from haplotypes determined manually from 454 reads. (Data for haplotypes inferred from genotypes reported in Table 1 in parentheses.)

M. sativa Subspecies	# of individuals	# of sequences	# of polymorphic sites	# of haplotypes	π	θ
CCoAoMT	Sequence length: 1340bp					
caerulea	27	51 (54)	9 (11)	16 (22)	0.0051 (0.0123)	0.0061 (0.0114)
falcata	35	65 (70)	15 (20)	23 (25)	0.0076 (0.0129)	0.01113 (0.015)
hemicycla	10	16 (20)	9 (14)	7 (10)	0.0101 (0.0127)	0.0095 (0.0117)
Overall	72	132 (144)	19 (21)	32 (42)	0.0077 (0.0116)	0.0124 (0.0158)
F5H exon 1	Sequence length: 723bp					
caerulea	25	41 (50)	23 (22)	21 (21)	0.0062 (0.0062)	0.0074 (0.0068)
falcata	32	54 (64)	38 (39)	31 (32)	0.0084 (0.008)	0.0121 (0.012)
hemicycla	8	15 (16)	16 (16)	10 (9)	0.0071 (0.0059)	0.0068 (0.0067)
Overall	65	110 (130)	51 (52)	54 (54)	0.0096 (0.0095)	0.0139 (0.0137)
*F5H*exon 2	Sequence length: 594					
caerulea	23	37 (46)	42 (42)	18 (18)	0.0159 (0.0144)	0.0177 (0.0169)
falcata	27	49 (54)	53 (52)	30 (29)	0.021 (0.0192)	0.0211 (0.0203)
hemicycla	7	11 (14)	32 (30)	6 (6)	0.0126 (0.0093)	0.0195 (0.0169)
Overall	57	97 (114)	58 (57)	46 (47)	0.0263 (0.0257)	0.0206 (0.0197)

Supplemental Table 4. Non-significant SNP-trait associations with P-values < 0.05.

Year	Gene	SNP	N	Marker effect			FDR *Q*-value
				F	*P*	*R2*	
2007	*F5H* exon 1	430	65	6.9959	0.0019	0.2252	0.1981
2007		430	65	6.6418	0.0025	0.2093	0.1981
2007		386	65	6.3806	0.0031	0.2011	0.1981
2007		430	65	6.1263	0.0038	0.1947	0.1981
2007		392	65	4.8933	0.0107	0.1542	0.4417
2007		392	65	4.414	0.0163	0.1403	0.4417
2008		175	65	4.0648	0.0222	0.1324	0.4417
2008		433	65	3.8881	0.0258	0.1198	0.4417
2007		175	65	3.8074	0.0278	0.1226	0.4417
2007		241	65	3.7493	0.0292	0.1207	0.4417
2008		50	65	3.6196	0.0328	0.1115	0.4417
2007		238	65	3.5976	0.0334	0.1158	0.4417
2008		241	65	3.4951	0.0368	0.1139	0.4417
2007		392	65	3.4787	0.0372	0.112	0.4417
2007		386	65	3.4073	0.0397	0.0964	0.4417
2008		238	65	3.3878	0.0405	0.1104	0.4417
2007		386	65	3.373	0.0409	0.1072	0.4417
2008		392	65	3.2626	0.0453	0.1063	0.4417
2007		241	65	3.2219	0.0469	0.1015	0.4417
2008		50	65	3.2217	0.047	0.105	0.4417
2007		386	65	3.2042	0.0476	0.1032	0.4417
2008		433	65	3.185	0.0486	0.1038	0.4417
2008	*F5H* exon 2	330	57	6.0997	0.0169	0.1087	0.7438
2008		276	57	3.6069	0.0197	0.1928	0.7438
2007		276	57	3.5792	0.0203	0.1981	0.7438
2008		345	57	4.1075	0.0224	0.1564	0.7438
2007		426	57	3.354	0.0262	0.1639	0.7438
2008		165	57	3.7964	0.0292	0.1304	0.7438
2008		432	57	3.7595	0.0301	0.1291	0.7438
2008		522	57	3.4984	0.0378	0.1133	0.7438
2007		345	57	3.3652	0.0425	0.1289	0.7438
2008		330	57	4.2205	0.0451	0.0692	0.7438
2008		78	57	3.2817	0.0458	0.1127	0.7438
2007		222	57	3.245	0.0477	0.1018	0.7438

				Marker effect			
Year	Gene	SNP	N	*F*	*P*	*R2*	FDR *Q*-value
2007	*Pal1* exon 1	251	67	5.5373	0.0062	0.1557	0.3205
2008		242	67	4.8632	0.011	0.134	0.3205
2007		305	67	4.5977	0.0139	0.1293	0.3205
2007		245	67	3.0957	0.0153	0.2176	0.3205
2007		254	67	3.338	0.0158	0.1877	0.3205
2008		338	67	3.7322	0.0159	0.1543	0.3205
2007		251	67	4.3936	0.0166	0.1378	0.3205
2008		80	67	5.9422	0.0177	0.0819	0.3205
2007		83	67	4.0918	0.0216	0.115	0.3205
2008		305	67	3.9492	0.0245	0.1088	0.3205
2007		323	67	3.9333	0.0248	0.1233	0.3205
2007		305	67	3.9088	0.0254	0.1226	0.3205
2008		242	67	3.7434	0.0294	0.1052	0.3427
2007		86	67	3.4187	0.0393	0.0961	0.3973
2008		323	67	3.4177	0.0393	0.0942	0.3973
2008		80	67	4.0133	0.0496	0.06	0.4409

				Marker effect			
Year	Gene	SNP	N	*F*	*P*	*R2*	FDR *Q*-value
2007	*Pal1* exon 2	1222	71	5.3256	0.0081	0.1623	0.6616
2007		856	71	7.4503	0.0082	0.1166	0.6616
2008		73	71	5.19	0.0082	0.1408	0.6616
2008		59	71	4.8919	0.0106	0.1444	0.6616
2008		118	71	4.8919	0.0106	0.1444	0.6616
2007		1222	71	4.9513	0.011	0.1387	0.6616
2008		505	71	6.81	0.0112	0.086	0.6616
2008		253	71	4.7901	0.0116	0.1299	0.6616
2007		1156	71	4.8066	0.0121	0.1509	0.6616
2008		49	71	4.6759	0.0128	0.138	0.6616
2007		1249	71	4.6659	0.0138	0.147	0.6616
2007		1219	71	4.6574	0.0139	0.1733	0.6616
2008		79	71	4.5846	0.0139	0.1244	0.6616
2007		1348	71	4.6654	0.014	0.1307	0.6616
2007		1156	71	4.5313	0.0153	0.1563	0.6616
2007		1324	71	4.4545	0.0165	0.1404	0.6616
2008		253	71	4.3828	0.0166	0.1156	0.6616
2008		328	71	4.3486	0.0169	0.1152	0.6616
2007		1222	71	4.4286	0.0171	0.1364	0.6616
2008		748	71	4.2235	0.0191	0.1135	0.6616
2007		856	71	5.7171	0.0198	0.0904	0.6616
2007		85	71	4.1562	0.0204	0.1054	0.6616
2008		337	71	4.08	0.0214	0.1066	0.6616
2007		826	71	5.5085	0.0222	0.0882	0.6616
2008		826	71	5.3819	0.0236	0.0851	0.6616
2007		433	71	3.9557	0.0241	0.1167	0.6616
2008		895	71	3.6419	0.032	0.1151	0.6616
2007		49	71	3.621	0.0326	0.1095	0.6616
2008		1156	71	3.6572	0.0327	0.1124	0.6616
2007		772	71	3.6146	0.0327	0.1164	0.6616
2007		43	71	3.5931	0.0334	0.1087	0.6616
2008		895	71	3.506	0.0361	0.1022	0.6616
2008		1348	71	3.5445	0.0362	0.1154	0.6616
2008		73	71	3.4751	0.0371	0.0991	0.6616
2008		328	71	3.4641	0.0372	0.0874	0.6616
2007		251	71	4.5254	0.0373	0.0685	0.6616
2007		778	71	3.4461	0.0383	0.0816	0.6616

				Marker effect			
Year	Gene	SNP	N	F	P	R2	FDR Q-value
2008		1084	71	3.0041	0.0388	0.1545	0.6616
2008		73	71	3.3935	0.0399	0.0933	0.6616
2008		79	71	3.3201	0.0427	0.0947	0.6616
2008		59	71	3.3167	0.0428	0.0875	0.6616
2008		118	71	3.3167	0.0428	0.0875	0.6616
2008		1348	71	3.3345	0.0436	0.1256	0.6616
2007		1084	71	2.881	0.0448	0.1608	0.6616
2008		769	71	3.2376	0.046	0.0938	0.6616
2008		253	71	3.2211	0.0467	0.0886	0.6616
2007		1324	71	3.2553	0.0467	0.1124	0.6616
2007		85	71	3.2157	0.0469	0.0973	0.6616
2008		328	71	3.195	0.0475	0.0859	0.6616
2008		1348	71	3.2002	0.0492	0.1143	0.6616
2008		61	71	3.1475	0.0499	0.0798	0.6616

Year	Gene	SNP	N	Marker effect			FDR *Q*-value
				F	*P*	*R2*	
	Comt exon						
2007	1	300	69	4.8138	0.0113	0.1327	0.6408
2007		275	69	2.7586	0.05	0.1003	0.6408
2007	*Comt* 2	583	69	7.8841	0.0072	0.1541	0.8656
2008		968	69	5.2001	0.0082	0.1441	0.8656
2007		675	69	4.5999	0.0151	0.1732	0.8656
2008		1160	69	5.4606	0.0231	0.0896	0.8656
2008		675	69	4.0847	0.0233	0.1305	0.8656
2008		675	69	3.9153	0.0269	0.1428	0.8656
2008		1037	69	3.8036	0.0277	0.1049	0.8656
2008		974	69	3.2488	0.0278	0.1341	0.8656
2007		923	69	4.7152	0.035	0.0921	0.8656
2008		877	69	3.487	0.0389	0.115	0.8656
2008		689	69	3.0299	0.039	0.1768	0.8656
2008		748	69	3.4726	0.0394	0.1145	0.8656
2008		1193	69	3.3667	0.0419	0.0982	0.8656
2008		550	69	3.3935	0.0422	0.1084	0.8656
2007		675	69	3.3696	0.0431	0.1266	0.8656
2008		675	69	3.2903	0.0462	0.1085	0.8656
2008		1037	69	3.2181	0.0468	0.0979	0.8656
2008		331	69	3.1975	0.0477	0.0864	0.8656
	Ccomt						
2007		793	71	8.1196	7.20E-04	0.1437	0.0597
2007		793	71	8.1194	7.20E-04	0.156	0.0597
2007		111	71	7.9494	9.01E-04	0.2151	0.0664
2008		111	71	7.2671	0.0016	0.202	0.101
2008		538	71	7.0863	0.0017	0.1507	0.101
2008		237	71	6.9286	0.002	0.1601	0.1054
2007		793	71	6.8164	0.0021	0.1469	0.1057
2007		113	71	4.826	0.0047	0.1939	0.2208

Supplemental Figure 1. Linkage disequilibrium for *F5H* exon 2, overall and by subspecies.

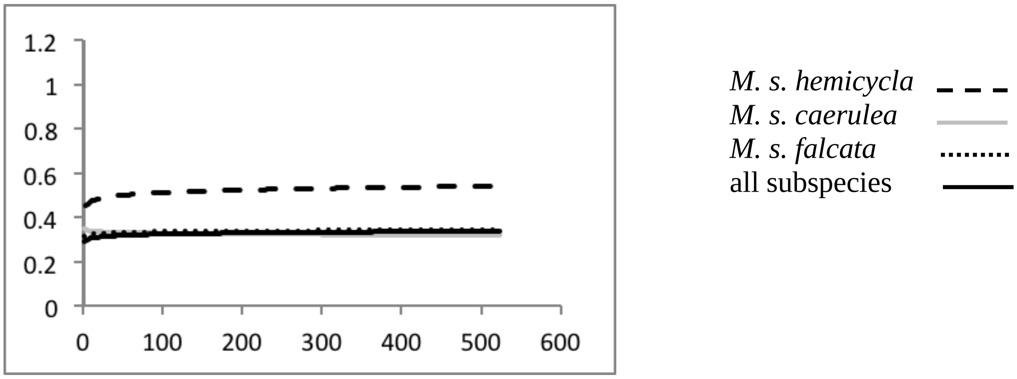

Supplemental Figure 2. Quantile-Quantile (QQ) plots to visualize the observed MLM *P*-value versus expected *P*-value distribution for *CCoAoMT* and *F5H* exon 2

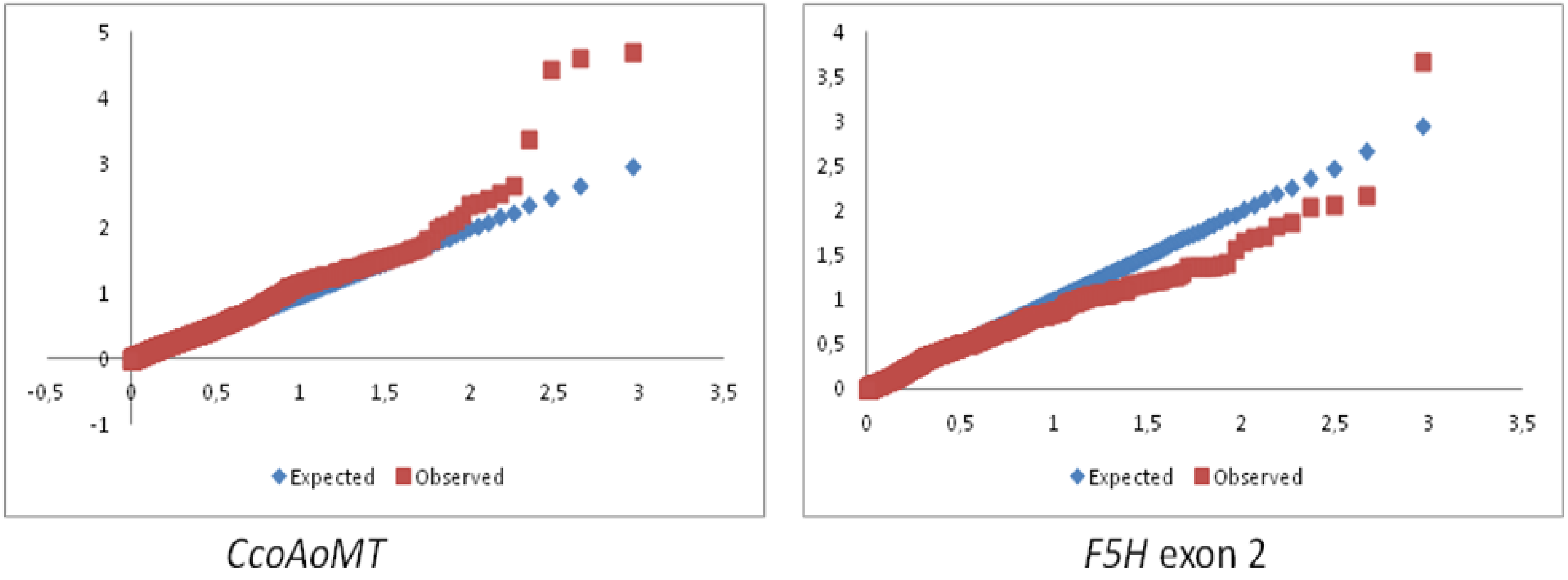

Supplement: Supplementary file 1 — Supplementary material 1 (PDF 495 kb) [file 122_2012_1854_MOESM1_ESM.pdf]
